# LAB-Secretome: a genome-scale comparative analysis of the predicted extracellular and surface-associated proteins of Lactic Acid Bacteria

**DOI:** 10.1186/1471-2164-11-651

**Published:** 2010-11-23

**Authors:** Miaomiao Zhou, Daniel Theunissen, Michiel Wels, Roland J Siezen

**Affiliations:** 1 Centre for Molecular and Biomolecular Informatics, Radboud University Nijmegen Medical Centre, PO Box 9101, 6500 HB Nijmegen, The Netherlands; 2 TI Food and Nutrition, Wageningen, The Netherlands; 3 NIZO food research, Ede, The Netherlands

## Abstract

**Background:**

In Lactic Acid Bacteria (LAB), the extracellular and surface-associated proteins can be involved in processes such as cell wall metabolism, degradation and uptake of nutrients, communication and binding to substrates or hosts. A genome-scale comparative study of these proteins (secretomes) can provide vast information towards the understanding of the molecular evolution, diversity, function and adaptation of LAB to their specific environmental niches.

**Results:**

We have performed an extensive prediction and comparison of the secretomes from 26 sequenced LAB genomes. A new approach to detect homolog clusters of secretome proteins (LaCOGs) was designed by integrating protein subcellular location prediction and homology clustering methods. The initial clusters were further adjusted semi-manually based on multiple sequence alignments, domain compositions, pseudogene analysis and biological function of the proteins. Ubiquitous protein families were identified, as well as species-specific, strain-specific, and niche-specific LaCOGs. Comparative analysis of protein subfamilies has shown that the distribution and functional specificity of LaCOGs could be used to explain many niche-specific phenotypes.

A comprehensive and user-friendly database LAB-Secretome was constructed to store, visualize and update the extracellular proteins and LaCOGs http://www.cmbi.ru.nl/lab_secretome/. This database will be updated regularly when new bacterial genomes become available.

**Conclusions:**

The LAB-Secretome database could be used to understand the evolution and adaptation of lactic acid bacteria to their environmental niches, to improve protein functional annotation and to serve as basis for targeted experimental studies.

## Background

Lactic Acid Bacteria (LAB) have been used for centuries in industrial and artisanal food and feed fermentations as starter cultures and are important bacteria linked to the human gastro-intestinal (GI) tract [1-8]. Phylogenetically they form a relatively compact group of mainly Gram-positive, anaerobic, non-sporulating, low G+C content acid-tolerant bacteria [9-12]. The genera that comprise the LAB belong to the order Lactobacillales, and are primarily *Lactobacillus, Pediococcus, Lactococcus*, *Streptococcus *and * Leuconostoc*, while some peripheral genera are *Enterococcus, Oenococcus, Aerococcus*, and *Carnobacterium*. Interestingly, even within such a compact group, vastly divergent phenotypes have been reported, providing indications of high flexibility and adaptation of these species to their living environments [13-16].

Extracellular and surface-associated proteins play a most important role in many essential interactions and adaptations of LAB to their environment [17-26]. By definition these proteins are either exposed on (anchored to membrane GO:0046658, intrinsic to external side of plasma membrane GO:0031233 and the cell wall, GO: 0005618) or released (extracellular milieu, GO:0005576) from the bacterial cell surface. On a genome scale these proteins form a subset of the proteome which contains both the exoproteome [27] and part of the surface proteome [28], but excluding the integral membrane proteins (GO: 0005887) and the proteins that are intrinsic to internal side of plasma membrane (GO:0031235). This subset of the proteome belongs to what Desvaux *et.al *have defined as "secretome" [27] and is known to mainly be involved processes such as: (1) recognition, binding, degradation and uptake of extracellular complex nutrients, (2) signal transduction, (3) communication with the environment and (4) attachment of the bacterial cell to specific sites or surfaces, e.g. to intestinal mucosa cells of the host [29-37]. Hence, genome-scale comparative analysis of these secretome (surface-associated and released from the cell) proteins may provide an understanding of the molecular function, evolution, and diversity of different LAB species and their adaptation to different environments.

Here we report a comparison of the predicted secretomes of 26 sequenced genomes of LAB representing 18 different species (Table 1). The secretome clusters of orthologous protein families (LaCOGs: Lactobacillales Cluster of Ortholog Groups) were extracted by combining homology clustering methods with protein subcellular location (SCL) prediction. The comparative analysis of LaCOGs shows many niche-specific protein families that can be used as leads for future experiments.

**Table 1:** The predicted LAB secretomes (genomes included in the original LaCOG analysis 43 are marked by *).

		Secretome proteins (%)							
LAB species and strains	Total proteins	A	B	C	D	E	F	G	Total (%)
*E.faecalis_V583*	3186	2.32	1.26	3.36	0.97	0.16	1.6	0.13	9.8
*L.acidophilus_NCFM*	1834	2.24	0.65	4.09	0.93	0	2.45	0.05	10.41
L.gasseri_ATCC_33323*	1733	1.85	0.69	3.92	0.52	0.12	0.69	0	7.79
L.johnsonii_NCC_533*	1789	2.07	0.89	4.3	0.56	0.39	0.06	0	8.27
*L.delbrueckii_bulgaricus* *_ATCC11842*	1536	1.56	0.13	3.45	1.04	0.07	2.02	0	8.27
*L.delbrueckii_bulgaricus* _ATCC_BAA-365*	1681	1.43	0.06	3.15	0.95	0.18	2.08	0	7.85
L.casei_ATCC_334*	2693	1.63	0.78	3.79	0.78	0.15	1.41	0.07	8.61
*L.casei_BL23*	2973	1.68	0.77	3.4	0.84	0	1.35	0.13	8.17
*L.salivarius_UCC118*	1973	0.91	0.25	3.4	0.61	0.15	1.27	0.1	6.69
*L.sakei_23K*	1845	1.52	0.33	3.36	0.76	0.05	2.06	0.27	8.35
L.plantarum_WCFS1*	2981	1.61	1.11	3.99	0.91	0.3	0.1	0	8.02
*L.brevis_ATCC_367*	2178	1.29	0.55	3.35	1.52	0.14	2.53	0.09	9.47
*L.fermentum_IFO_3956*	1826	0.66	0.22	2.96	0.55	0	1.15	0.05	5.59
*L.helveticus_DPC_4571*	1597	1.38	0.13	4.51	0.44	0	2.13	0	8.59
*L.reuteri_F275_JGI*	1881	0.74	0.21	3.67	0.85	0	1.01	0	6.48
*L.reuteri_F275_Kitasato*	1803	0.78	0.28	3.55	1	0	1.22	0	6.83
*L._lactis_cremoris_MG1363*	2393	1.46	0.46	3.01	0.79	0	1.96	0	7.68
L.lactis_cremoris_SK11*	2459	1.38	0.41	3.17	1.02	0.12	1.67	0.08	7.85
L.lactis_lactis_IL1403*	2284	1.4	0.61	4.29	0.74	0.04	1.62	0.18	8.88
*L.citreum_KM20*	1784	0.06	0.28	4.43	1.23	1.23	0	0.06	7.29
S.thermophilus_CNRZ1066*	1872	1.28	0.05	3.47	0.53	0.27	0.43	0.05	6.08
S.thermophilus_LMD-9*	1669	1.5	0.24	3.89	0.54	0.18	0.84	0	7.19
*S.thermophilus_LMG_18311*	1854	1.29	0.11	3.78	0.54	0.49	0.65	0	6.86
L.mesenteroides_ATCC_8293*	1966	0.1	0.31	4.93	1.12	0.31	1.22	0.15	8.14
O.oeni_PSU-1*	1664	0.12	0.06	4.33	0.9	1.56	0	0.06	7.03
P.pentosaceus_ATCC_25745*	1727	1.1	0.17	3.88	0.35	0.17	0.98	0.12	6.77

A: Lipid anchored; B: LPxTG Cell-wall anchored; C: N-terminally anchored (No cleavage site); D: N-terminally anchored (with cleavage site); E: Secreted via minor pathways (bacteriocin) (no cleavage site); F: Extracellular (with cleavage site); G: C-terminally anchored (with cleavage site)

The SCL prediction was made by LocateP.

The complete results of this study are stored in our open-source database LAB-Secretome http://www.cmbi.ru.nl/lab_secretome with a user-friendly web-interface. An automatic update scheme was constructed to be able to add information to the database on new bacterial genomes.

## Results and Discussion

### Construction of the secretome protein clusters (LaCOGs)

In this study we focus on those proteins that are predicted to be wholly or largely on the outside of the cell, regardless of the translocation systems. These proteins form a sub-proteome of what Desvaux *et.al *defined as the "secretome" [27] by excluding the translocation systems, the integral membrane proteins, and non-protein products. Although we adapt this term "secretome" to describe our protein subset of interest, we must specify that in our analysis the term "secretome" refers to only the proteins that are released from the cells to the extracellular milieu (also called exoproteome), and the proteins that remain cell-surface associated, but nothing else.

**Figure 1:**
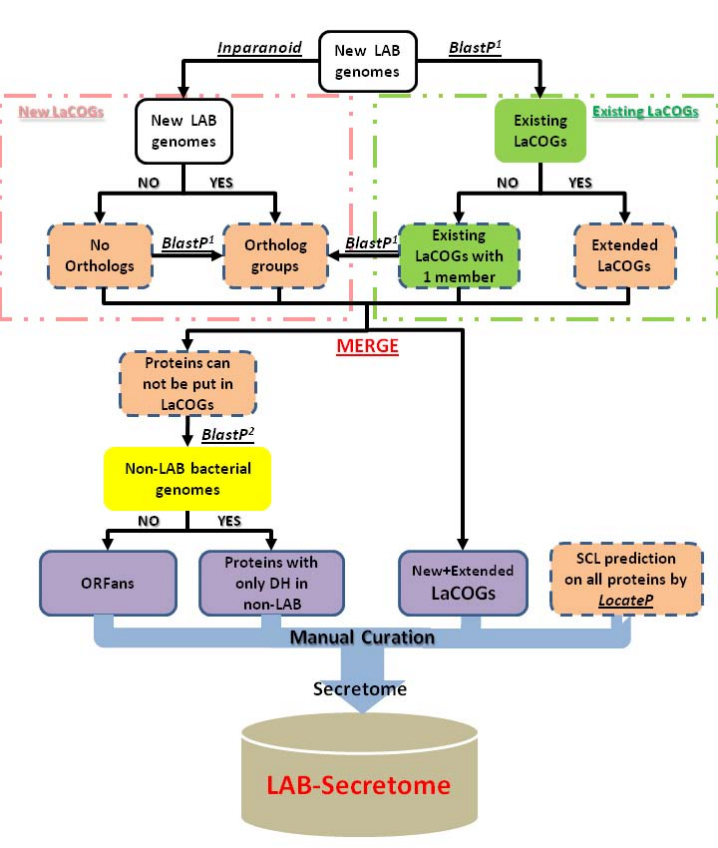
**The flowchart for constructing the secretome LaCOGs**. The completely sequenced LAB genomes are used as input data. No plasmid sequences were used for the Inparanoid search. The squares with dash-line frames are intermediate products that are not user-queryable from the LAB-Secretome interface; the squares with full-line frames are the final information stored in LAB-Secretome database. The upper left frame shows the processes that produce new LACOGs; the upper right frame shows the processes that extend existing LaCOGs. The new LaCOGs are coded starting with "9", the extended existing LaCOGs retain the original names from Makarova *et.al *[43]. BlastP^1^: the Blast results were processed by a revised criterion "uniform top 3" (see Material and Methods); BlastP^2^: the Blast results were processed by cut-off of 1e-3 and aligned sequence coverage of 60% for distant homolog identification. This work scheme can be used to update the LAB-Secretome database when new bacterial genomes are available.

Ideally, a comparative secretome analysis should be performed on the experimentally validated sub-proteomes or on *in silico *predicted secretome proteins with the highest possible accuracies. However, it is well-known that wet-lab proteomic studies are extremely costly and can lead to many false predictions of subcellular location, while all the currently available *in silico *protein SCL predictors have only 80%-93% prediction accuracy [38-41]. Therefore, instead of clustering predicted extracellular proteins directly, we designed an alternative process which firstly groups all proteins in the sequenced LAB genomes into ortholog groups (LaCOGs) and afterwards extracts the secretome groups by using genome-scale SCL predictions (Figure 1). In this way, the wrongly predicted secretome proteins could be reduced because homologous proteins with similar functions and domains always tend to have the same SCL, and *vice versa *[39-42].

**Figure 2:**
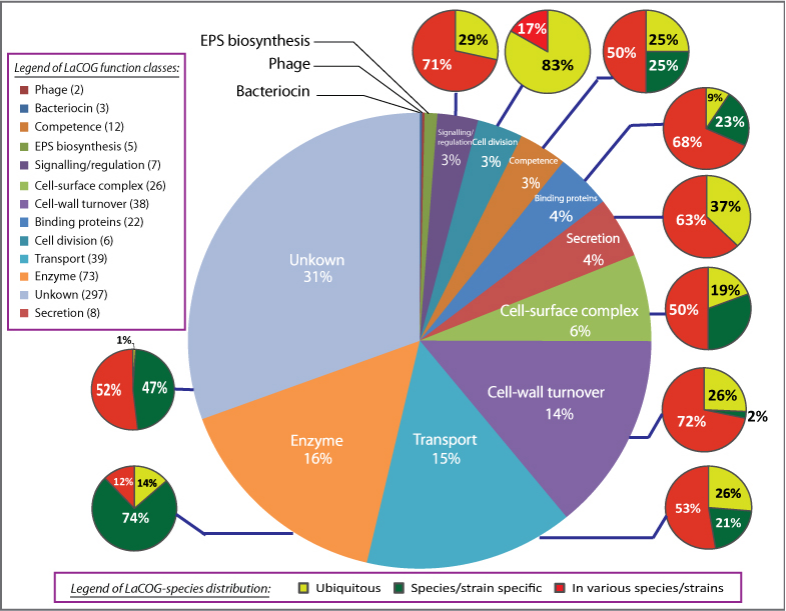
**overview of distribution of secretome proteins in LaCOGs**. The central pie depicts the distribution of secretome proteins in LaCOGs according to their functional classes. The percentage was calculated as the number of proteins in the category divided by the total of 3357 secretome proteins that were clustered into LaCOGs. The number of LaCOGs in each category is listed in the pie chart legend behind the name of the functional class. The separate yellow-red-green piecharts for each functional class represents the distribution of this LaCOG in the LAB genomes, i.e. ubiquitous, .species/strain-specific, or variable.

The *Lactobacillales*-specific clusters of orthologous groups of proteins (LaCOGs) previously generated by Makarova *et.al *[43] were used as the basis for protein clustering into protein families. In total 3374 (729 new and 2645 existing) LaCOGs were formed by adding 14 recently sequenced LAB genomes to the Makarova et. al. set. Subsequently, a genome-scale SCL prediction was performed on all proteins in the 26 genomes (Table 1). By combining the SCL prediction and LaCOGs, and after manual curation (see below), we defined 462 secretome LaCOGs (of which 212 are new compared to the Makarova et. al. set) composed of 3357 proteins, representing 7.4% of the complete genome dataset and 93% of all predicted secretome proteins in these 26 genomes. We defined thirteen general functional classes for these proteins, and the distribution of these clustered secretome proteins over the classes and LaCOGs is shown in Figure 2. An additional 249 putative secretome proteins could not be grouped into these LaCOGs, comprising 69 proteins that had only a distant homolog in non-LAB, and 180 proteins that had no homolog in any sequenced bacterial genomes, which we termed the extracellular "ORFans" (Table 2, Additional file 1, sheet S1).

**Table 2:** Overview of the LaCOGs (genomes included in the original LaCOG analysis 43 are marked by *).

LAB species and strains	Secretome size	Proteins in LaCOG	Distant Homologs	ORFans	LaCOGs
*E.faecalis V583*	281	232	22	27	131
*L.acidophilus NCFM*	171	161	2	8	108
*L.brevis ATCC 367*	177	154	5	18	113
L.casei ATCC 334 *	192	187	3	2	148
*L.casei BL23*	205	197	0	8	153
*L.citreum KM20*	112	112	0	0	93
L.delbrueckii bulgaricus ATCC BAA-365 *	115	113	0	2	94
*L.delbrueckii bulgaricus ATCC11842*	87	79	3	5	68
*L.fermentum IFO 3956*	112	112	0	0	89
L.gasseri ATCC 33323 *	115	113	0	2	88
*L.helveticus DPC 4571*	131	123	2	6	97
L.johnsonii NCC 533 *	236	209	6	21	131
*L.lactis cremoris MG1363*	105	103	0	2	86
L.lactis cremoris SK11 *	105	105	0	0	87
L.lactis lactis IL1403 *	136	114	4	18	80
L.mesenteroides ATCC 8293 *	112	94	5	13	77
L.plantarum WCFS1 *	160	151	5	4	123
*L.reuteri F275 JGI*	159	156	1	2	124
*L.reuteri F275 Kitasato*	171	156	2	13	123
*L.sakei 23K*	114	103	4	7	80
*L.salivarius UCC118*	135	126	3	6	103
O.oeni PSU-1 *	95	90	0	5	70
P.pentosaceus ATCC 25745 *	99	89	1	9	79
S.thermophilus CNRZ1066 *	90	90	0	0	77
S.thermophilus LMD-9 *	97	94	1	2	84
*S.thermophilus LMG 18311*	94	94	0	0	81

**Figure 3:**
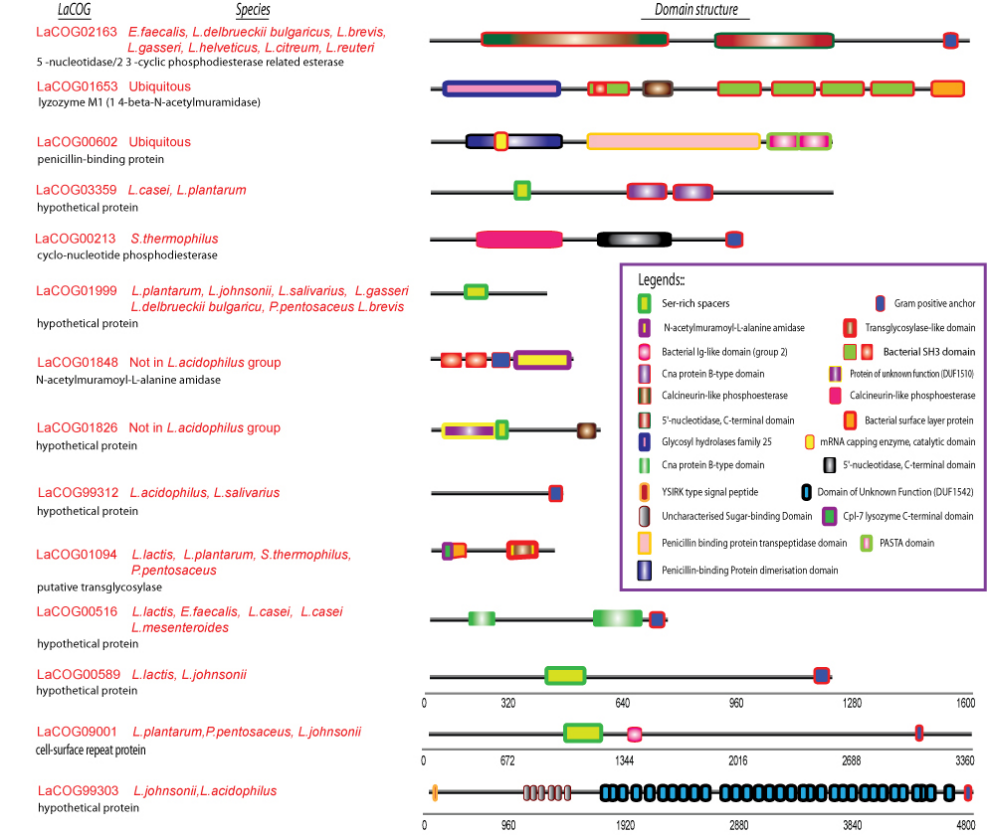
**Variations in domain composition**. Examples of LaCOGs families showing different domain types, domain compositions and repeats.

Although the LAB genomes vary in size, the size of the secretome as a fraction of each genome was fairly consistent (6-10%), as well as the distribution of proteins over different SCLs. The N-terminally anchored proteins with no signal peptidase cleavage site are the most abundant kind among all predicted secretome proteins. A striking feature of numerous secretome proteins, and particularly surface-associated proteins, is that they are large and consist of many different domains (often in repeats), and domain compositions (see examples in Figure 3). In fact, this variation in domain composition has been used in constructing and sub-dividing the LaCOGs and separating sub-families of homologous proteins. Distinct combinations of domains provide hints for functions of these extracellular proteins in cell-wall metabolism, cell-wall binding and their communication with the environment (see below).

### False predictions and pseudogenes

The preliminary secretome clusters were curated manually and corrected based on expert knowledge, e.g. for false-positive and false-negative predictions, incorrect gene starts, pseudogenes, etc. Examples of proteins of known intracellular function, but with consistent false-positive extracellular SCL prediction are listed in Additional file 2, sheet S1. In most cases the mis-prediction was caused by an α-helix-like N-terminal sequence in these proteins (possibly as part of the hydrophobic core of a globular protein), leading to the prediction as a signal peptide by LocateP. A further improvement was made by finding and removing those LaCOGs that have proteins which are anchored in the cell membrane with a single N-terminal transmembrane helix, but with the rest of the protein inside the cell (so-called outside-in topology, GO:0031235) [44-53]. By aligning proteins within these LaCOGs we found that these proteins do not have positively charged residues preceding the N-terminal hydrophobic helix, but exclusively have a positively charged residue(s) immediately downstream of the transmembrane helix (examples in Additional file 2, sheet S2). Hence such features could be used for further development of a model for SCL prediction of N-terminally anchored proteins by LocateP.

Nearly 400 pseudogenes were identified, but this is probably an underestimate. In most cases this was due to gene frameshifts, and occasionally to N- or C-terminal truncation of genes. Most of these genes could be concatenated to encode larger proteins with high similarity to known proteins in the LaCOGs. Many of these pseudogenes were initially predicted to encode intracellular proteins by LocateP, but after concatenation these proteins are predicted to be extracellular and/or contain domains of extracellular functionalities. An example are the proteins encoded by adjacent genes LSA1731 and LSA1730 in *L.sakei *23K which were annotated as hypothetical proteins. The concatenated protein showed high similarity to proteins in LaCOG02935 which were exclusively cell-surface protein Csc complex family members [54]. In total 129 concatenated pseudoproteins were made with 279 protein fragments (Additional file 3, sheet S1), while 87 pseudogenes could not be combined (Additional file 3, sheet S2).

### The LAB-Secretome database

**Figure 4:**
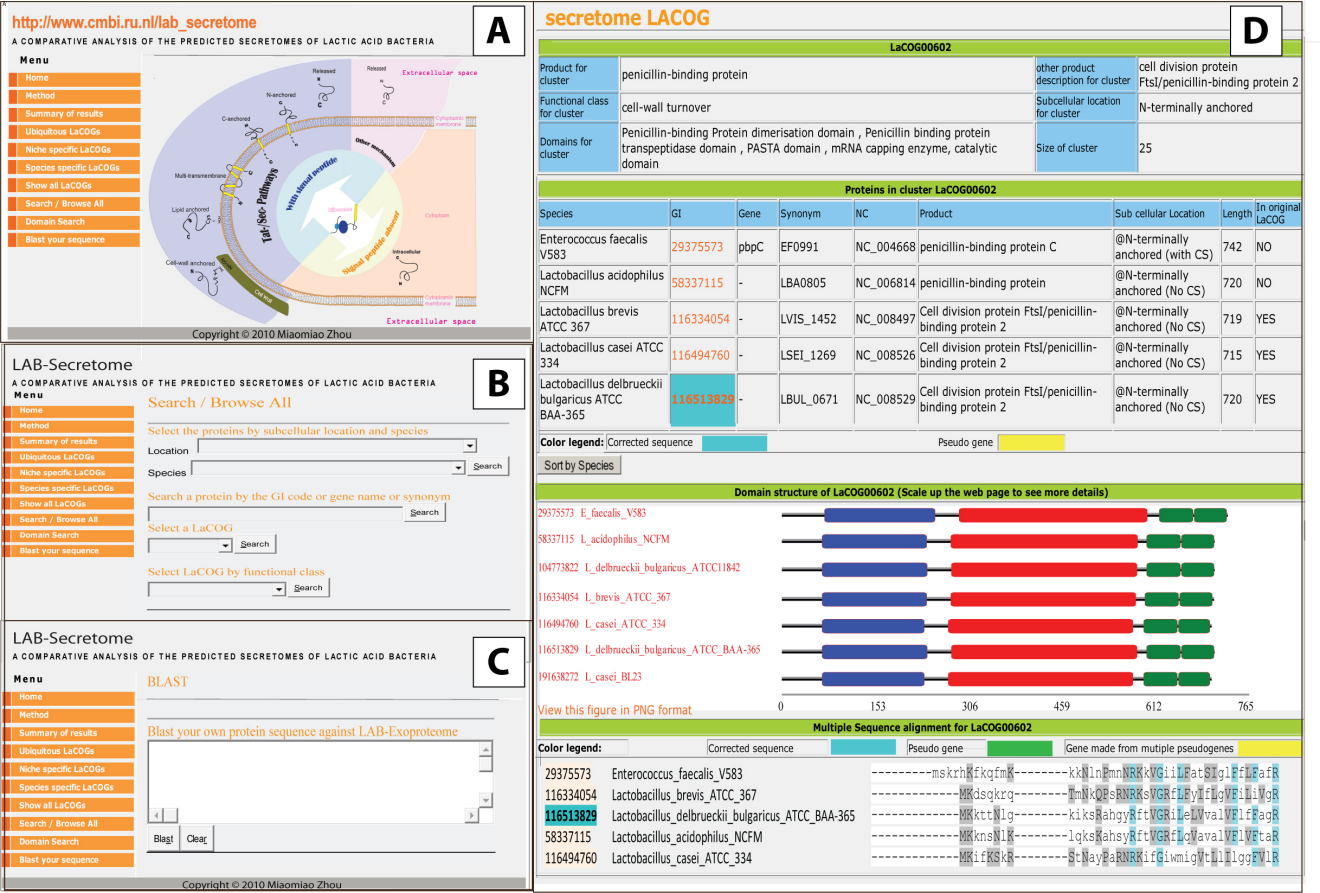
**Screen shot of the LAB-Secretome database**. A: Overview page of the database showing statistical information of the predicted LAB secretomes with active links to their corresponding pages; B: The search engine in LAB-Secretome which can browse the database by various types of queries; C: The BlastP search page of LAB-Secretome; D: An example page depicting parts of the detailed information that LAB-Secretome presents for each LaCOG.

The LAB-Secretome database http://www.cmbi.ru.nl/lab_secretome was constructed to store and browse all the predicted extracellular proteins and LaCOGs. An overview page summarizes all predicted secretomes, LaCOGs, distant homologs in non-LAB species and the ORFans, with hyperlinks to the corresponding HTML pages to help users to browse the whole database (Figure 4A). The LAB-Secretome database can be queried in many ways, e.g. by bacterial species, protein subcellular location, protein accession identifiers, LaCOG numbers, protein functional classes, and Pfam domain accession codes or domain functions (Figure 4B). Visualization includes a description of LaCOG members and function, protein functional domain composition, and multiple alignments with notification of corrected start codons, pseudogenes and concatenated proteins (Figure 4D). A Blast function, utilizing the BlastP [55] program, enables users to query the clustering information of their proteins of interest to the extracellular proteins and families that are already in the database (Figure 4C). An automatic updating scheme for the LaCOGs (Figure 1) was designed to ensure that the need for manual curation is minimized when adding new bacterial genomes to the database.

### Overview of the extracellular protein families

#### Ubiquitous/essential LaCOGs

Only 22 LaCOGs were found to be fully conserved among all 26 LAB secretomes, or only lacking in 1 genome (5 LaCOGs), e.g. the absence of an ATP-dependent protease from LaCOG01453 in *P. pentosaceus *(Additional file 1, sheet S3).

Most of these LaCOGs contain proteins with universal functionalities involved in cell-wall metabolism, secretion, transport and DNA uptake (Figure 2). Only one conserved family (LaCOG01219) contains proteins of as yet unknown function, but presumably essential as they are conserved in all genomes.

#### Most common functionalities in the secretomes of LAB

**Figure 5:**
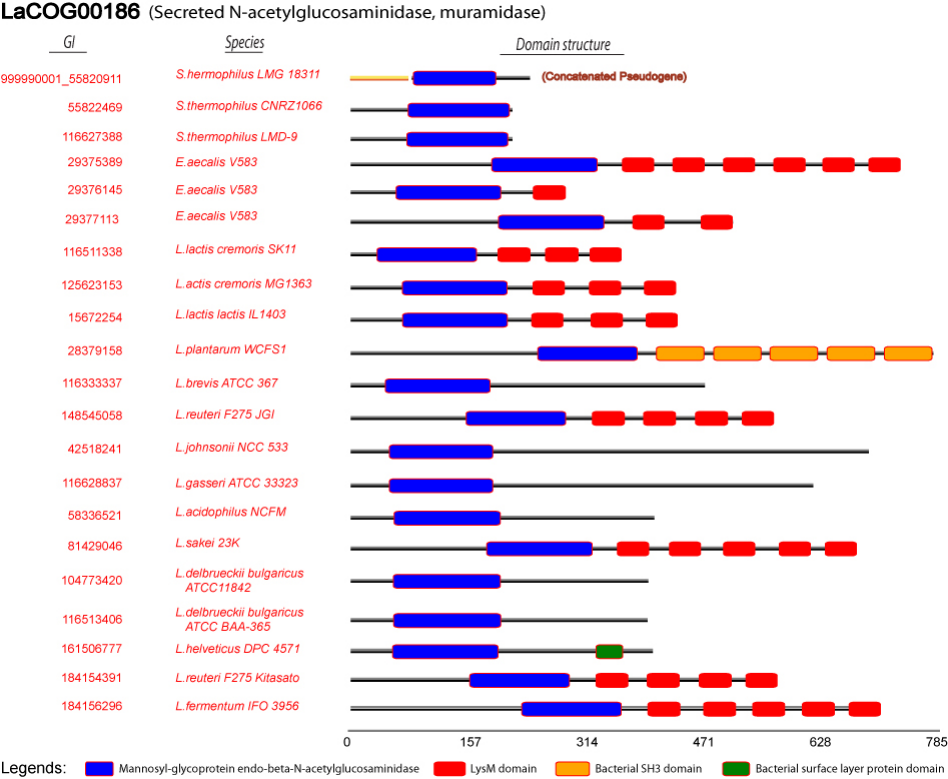
**Domain structure variation of enzymes within a family**. Examples of an enzyme family (N-acetyl-glucosaminidase) with variations in the type and number of cell-envelope binding domains.

Among all 215 secretome LaCOGs with known or presumed functions, almost half of them contain proteins which are involved in cell-wall metabolism, e.g. the muramidase, lysin, lysozyme and beta-lactamase families (Figure 2). Many of these enzyme families are further subdivided into different LaCOGs based on variations in sequence homology and protein domain compositions, and some may represent species/niche-specific subfamilies. One example is the subdivision of proteins with an Nlpc/P60 family domain (e.g. gamma-D-glutamate-meso-diaminopimelate muropeptidase) into 5 separate LaCOGs (Additional file 4, sheet S1). These proteins vary in length from ~150 to ~500 amino acids, all with the Nlpc/P60 domain in the C-terminal part. In only one of these subfamilies (LaCOG90015), all 16 members have 1-3 copies of LysM domains (Pfam PF01476) in their N-terminal part, indicating extra binding functions to the cell-envelope. A similar domain architecture is found in one of the four N-acetylmuramoyl-L-alanine amidase subfamilies (LaCOG01848), which has an enzymatic C-terminal domain and 0-3 N-terminal SH3 domains (Pfam PF08239), known to bind to proline-rich regions of proteins. In the pepdidoglycan hydrolase subfamilies LaCOG00186 and LaCOG01653 the enzymatic domain is located at the N-terminus and can be followed by different kinds, combinations and numbers of binding domains such as LysM, SH3 or surface layer domain (Pfam PF03217) (Figure 5). These examples all illustrate that the many types of extracellular enzymes involved in cell-wall turnover have different mechanisms to attach to components of the cell surface.

#### Niche-specific LaCOG families

##### 1/*L. acidophilus *complex specific

The *acidophilus *"complex" including the species *L. acidophilus, L. johnsonii, L. gasseri, L. delbrueckii ssp bulgaricus *and *L. helveticus *has long been regarded as a phylogenetic subgroup [56-58]. About 30 LaCOGs appear to be specific for these species (Additional file 1, sheet S4). Their proteins include an ABC-type phosphate/phosphonate transport system (LaCOG02118), the aggregation promoting factor (LaCOG90005) [59-61], a putative competence protein (LaCOG03110) and several families of S-layer proteins, which may reflect the special binding function that these S-layer proteins generally share in these *acidophilus *complex species [62-69]. Interestingly, twenty of these *acidophilus *complex-specific LaCOGs contain only extracellular proteins of unknown function, and it should be challenging to focus on experimental determination of their function.

##### 2/GI-tract specific

If we consider the LAB species *L. acidophilus*, *L. johnsonii*, *L. gasseri*, *L. reuteri*, and *L. salivarius *to be specifically found in the GI-tract, then we can identify 17 LaCOGs which are not found outside of this group, of which 13 families contain only proteins of unknown function (Additional file 1, sheet S4). One mucus-binding protein family (LaCOG02280) was found to be specific for these GI-tract LAB, and contains 4 proteins from *L. acidophilus, L. gasseri *and *L. johnsonii*. All four proteins are larger than 2300 amino acids, contain a signal peptide with YSIRK domain (Pfam PF04650) and appear to be anchored to the peptidoglycan by an LPxTG cell-wall anchor (Pfam PF00746). Each protein has 5-11 copies of a mucus-binding domain, as defined by Boekhorst et al [60], showing their particular role in binding to mucus components in the GI-tract [5,70-72]. The 3 D structure of this domain of 184 residues has recently been determined and shows similarity to the functional repeat found in a family of immunoglobulin-binding proteins [73].

##### 3/Plant-associated specific

Twelve LaCOGs appear to be specific for the group of plant-associated species *Leuconostoc, Oenococcus*, *L. plantarum, L. brevis*, and *P. pentosaceus*, of which 7 familes contain only proteins of unknown function (Additional file 1, sheet S4). One of these (LaCOG02876) includes 4 homologous proteins from *L.brevis*, *L.plantarum, O.oeni *and *L. citreum*, which show a high sequence similarity to each other, but the protein from *L. plantarum *has a much longer serine-rich spacer between the N- and C-terminal domains. A similar domain structure differing in a long serine-rich spacer is seen in the 2 hypothetical proteins from *L. plantarum *and *L. brevis *in LaCOG02927.

##### 4/Dairy LAB specific

A few protein families were found only to occur in the secretomes of the dairy LAB *S. thermophilus, L. lactis and E. faecalis *(Additional file 1, sheet S4). These proteins have functional properties that may be relevant to the dairy niche, e.g. LaCOG00374 contains ABC transporter substrate-binding proteins for polar amino acids, and could possibly be required for growth in milk [74-77]. The *L. lactis *strains have a single copy of this gene, while the *S. thermophilus *strains all have 3 consecutive genes encoding paralogs of this amino acid-binding protein. All dairy *Streptococcus *and *Lactococcus *strains contain a single gene encoding a beta-lactamase (LaCOG00012) which may play a role in destroying penicillin that these strains may encounter in milk [78-82]. A putative chitinase (glycosyl hydrolase family 18; LaCOG02690) is found exclusively in *E. faecalis *and in *L. lactis *strains.

#### Species-specific and strain-specific LaCOGs

Up to 150 LaCOGs were found to be species-specific or strain-specific (Additional file 1, sheet S5). The distinction is not so clear yet because for some species several strains were sequenced (e.g. *L. lactis, S. thermophilus*) while for many species only a single strain was sequenced to date. Most of these families are made up solely of hypothetical proteins with highly conserved sequence (Figure 2). *L. casei *and *L. lactis *have the highest number of species-specific LaCOGs, indicating that they may have more unique extracellular functions. Examples of species-specific extracellular proteins are the PrgA/PrgB/PrgC surface proteins of *E. faecalis *[83-85], an alpha-amylase (LaCOG02644) in *L. lactis *strains, a phospholipase A2 family enzyme (LaCOG99223) in *L. casei *strains, a cyclo-nucleotide phosphodiesterase (LacOG00213) in *S. thermophilus *strains, and a mucus-binding protein (LaCOG90010) in *L. delbrueckii *strains.

#### Extracellular proteins not in LaCOGs: ORFans and proteins with only distant homologs in non-LAB

About 249 putative extracellular proteins could not be classified into LaCOG families, and comprise 69 proteins that have only distant homologs in non-LAB species and 180 ORFans that are species-specific (Additional file 1, sheets S6 and S7). While the ORFans are nearly all hypothetical proteins of unknown function, the distant homologs also contain proteins with a variety of known functions, such as extracellular enzymes (e.g. xylanase, pectate lyase, endo-beta-N-acetylglucosaminidase, proteases and beta-fructosidase), substrate-binding proteins of transporters, miscellaneous binding proteins and specific bacteriocins. The uniqueness of these proteins suggests that most species or strains have a few unique extracellular proteins that are not found in other sequenced LAB, and may encode unique functions that are related to their environmental niche. Quite a few of the proteins of unknown function are predicted to be lipid-anchored and therefore may represent substrate-binding proteins of uncharacterized transporters.

### Specific enzyme families

LAB possess a variety of extracellular hydrolytic enzymes and transglycosylases which presumably relate to interactions with their environment, e.g. for degradation of growth substrate polymers. These enzymes have been clustered and sub-divided into protein families (LaCOGs) based on specific domain compositions (Table 3, Additional file 4, sheet S2). For instance, the subtilisin-like serine proteases (Pfam PF00082), known to be important for growth on protein substrates [86-89], were clustered into 2 LaCOGs: the first family (LacOG02153) is composed of 7 proteins containing a protease-associated PA domain (Pfam PF02225) inserted in the catalytic domain which forms a lid structure that covers the active site, whereas the other family (LaCOG90024) was only found in *L. casei *and *L. acidophilus*, and contains subtilisin-like serine proteases without the PA domain. Putative transglycosylases, also referred to as aggregation-promoting factors [59,90-92], are divided into three subfamilies (LaCOG01580, LaCOG02932, LaCOG90005), and have a highly conserved C-terminal domain [71]. Furthermore, there are several families of hydrolases of unknown function (Table 3). The extracellular alpha/beta hydrolases with a DUF915 domain (Pfam PF06028) are subdivided into four families, two of which are highly populated (LaCOG01137 and LaCOG01138, with 46 and 30 members, respectively) and found in nearly all LAB, suggesting that they have an essential, but as yet unknown, function.

**Table 3:** Examples of specific enzyme and binding-protein sub-families

Product	LaCOG	Functional domain	Distribution	Special features
**Specific enzyme families**				

Subtilisin-like serine protease	LaCOG02153	Subtilase family	*L. casei, L. delbrueckii bulgaricus*, *L. johnsonii, L. lactis, S. thermophilus*	PA domain (PF02225) inserted in the subtilase family domain
	LaCOG90024	Subtilase family	*L. acidophilus*, *L. casei*	no PA insert domain

Trans-glycosylase	LaCOG01094	Transglycosylase-like domain,	mainly in *L.plantarum*, *L.lactis, S.thermophilus*	different domains for PG binding
	LaCOG01589	aggregation promoting factor related surface protein	not in *L.acidophilus *group	PG bound by LysM domain; highly conserved C-terminal domain ending in GWY
	LaCOG02932	aggregation promoting factor related surface protein	only in *L.delbrueckii bulgaricus*, *L.plantarum*, *L.acidophilus *group	highly conserved C-terminal domain ending in WY
	LaCOG90005	aggregation promoting factor related surface protein	only in *L.acidophilus *group	highly conserved C-terminal domain ending in GWY

Dextran sucrase	LaCOG90016	glycosyl hydrolase family 70	only in *Leuconostoc*, *L. reuteri*, *O. oeni*	

**Cell-surface hydrolases**				

alpha/beta hydrolase	LaCOG01137	alpha/beta hydrolase of unknown function (DUF915)	ubiquitous	
	LaCOG01138	alpha/beta hydrolase (DUF915)	Ubiquitous	
	LacOG01920	alpha/beta hydrolase (DUF915)	only in *L. delbrueckii bulgaricus*, *L.plantarum *, *L.casei*	
	LaCOG02785	alpha/beta hydrolase (DUF915)	only in *L.plantarum *, *L.casei *, *L.sakei*	

lipase/Acyl-hydrolase	LaCOG00342	GDSL-like Lipase/Acylhydrolase	not in *L.acidophilus *group	with GDSL-like motif

general cell surface hydrolase	LacOG02019	cell surface hydrolase membrane-bound (putative)	only in *L.delbrueckii bulgaricus*, *L.plantarum*,*L.casei *, *L.fermentum*	
	LaCOG01618	cell-surface hydrolase;	only *in L.plantarum *, *L.delbrueckii bulgaricus*, *P.pentosaceus*	

**Binding proteins**				

mannose-specific adhesion	LaCOG01741	MUB domain, Gram positive anchor	only in *L.plantarum*, *L.delbrueckii bulgaricus*, *P.pentosaceus*, *L.acidophilus *group	

collagen-binding protein	LaCOG00092	Collagen binding domain, Gram positive anchor	not in *L.acidophilus *group	

mucus-binding protein	LaCOG00885	MucBP domain (Classical), Gram positive anchor	not in *L.acidophilus *group	Leucine Rich Repeat, PT repeat
	LaCOG01470	MUB domain, Gram positive anchor		many pseudogenes, most *L.acidophilus *group proteins have YSIRK-type signal peptide
	LacOG02280	MUB domain, Gram positive anchor	only in *L.acidophilus *group	very large, YSIRK-type signal peptide
	LaCOG03211	MUB domain, Gram positive anchor		5 of 10 are pseudogenes; YSIRK SP in *L.acidophilus *group members
	LacOG99309	MUB domain, Gram positive anchor	only in *L.acidophilus *group	all pseudogenes; YSIRK type signal peptide

chitin-binding protein	LaCOG01300	Chitin binding domain	*E.faecalis*, *L.plantarum*, *L.sakei*, *L.lactis*	maybe related to niche

adherence protein	LaCOG01366	von Willebrand factor type A domain, Cna protein B-type domain	only in *L.lactis*, *E.faecalis*, *L.citreum*, *L.casei*	

### Specific binding-protein families

Many extracellular proteins contain known domains for binding to macromolecular substrates. In addition to domains for binding to the cell wall of the producing cell (e.g. LysM, SH3), several other domains are found which are related to binding to host macromolecules (e.g. domains annotated as mucus-binding, chitin-binding, collagen-binding, fibronectin-binding, carbohydrate-binding, etc) (Table 3). Some of these annotations derive from *in vitro *binding studies and may not reflect *in vivo *functions. In LAB, mucus-binding domains (MUB, MucBP) are found in many proteins and are thought to play a role in binding to the host GI-tract mucus layer [57,93,94]. An enormous variety is found in the size of these mucus-binding proteins and in the number of mucus-binding domains. We have made a preliminary separation into 7 different subfamilies of mucus-binding proteins based on protein size, sequence homology, domain composition and phylogeny (Table 3). The three largest subfamilies are (1) LaCOG00885 containing 11 members from different LAB but not from *L. acidophilus *group members, (2) LaCOG01470 with 28 members, found in many LAB, and (3) LaCOG03211 which includes 10 proteins. The proteins of LaCOG00885 contain solely the MucBP domains as defined by Pfam (PF00746), while the proteins of the other two LaCOGs possess multiple copies of the larger MUB domains as defined by Boekhorst *et al. *[71] (see also Figure 2 in[95]). Many mucus-binding proteins of *L. acidophilus *group members contain an N-terminal [Y/F]SIRKxxxGxxS-containing signal peptide (PF04650) which was earlier reported as a typical characteristic of the *L. acidophilus *MUB proteins [94,96], and may relate to a specific function in sorting or folding [97,98]. Furthermore, it is striking that many large genes encoding mucus-binding proteins are pseudogenes (e.g. in LaCOG01470, LaCOG03211 and LaCOG99309). While it is unlikely that these are all due to sequencing errors, it is not clear yet whether these are truly pseudogenes, or possibly may encode functional proteins after transcription with strand-slipping [5,71].

## Conclusions

Lactic Acid Bacteria (LAB) occur naturally in many different fermentation environments such as plant, meat, dairy and cereal. Overall similarities have been identified among the genomes of many LAB species [61,99-105]. However, bio-diversity has also been reported frequently, showing that subtle variations in presence or absence of proteins and functional domain composition might lead to important traits during bacterial adaptation to their living environments [106-113]. Our comparative research on extracellular and surface-associated protein families has provided a more solid basis for this hypothesis. Universal families have been identified which are apparently essential for survival of all LAB, but also species-specific protein families. Besides the clustered proteins with known functions, many families of hypothetical proteins and unique proteins (ORFans and proteins with only distant homologs in non-LAB) were found.

Protein clustering supports niche-dependent features of specific subgroups of LAB (e.g. the *L. acidophilus *group) and could aid in linking bacterial phenotypes to genotypes. The distinct sub-families of the different LaCOGs have provided clues for adaptation of the bacterial cells to their living environment, such as the GI-tract. The result of this study can be used as leads for experimental work on the molecular evolution, diversity, function and adaptation of bacteria to specific environments.

Our clustering methods and database structure were designed in a way that allows adoption to other groups of bacteria than LAB. The analysis results are stored in a queryable database which provides vivid browsing functions for users, and will be updated regularly to guarantee the continuation of the service to the biology community. Our clustering information into families could definitely help in checking the quality of newly sequenced genomes and for genome (re-)annotation.

## Methods

### Genome sequences and bioinformatics tools used in this research

The genome sequences of 26 selected representative lactic acid bacteria, including the protein functional annotation and the gene contexts, were obtained from the NCBI bacterial genome database (version 15 Aug., 2008) [114].

BlastP (default cutoff values of E < 1, low-complexity filter disabled) [55] and Inparanoid [115] were used for sequence homology and orthology searches, respectively. Protein subcellular location (SCL) was predicted by LocateP [38]. Multiple sequence alignments were constructed using Muscle [116]. Motif searches were performed using MEME and MAST [117]. Protein domains (version Dec. 2008) [118] originating from the Pfam database [119-121] and additional HMMs reported in other studies [54,71,96,122-124] were searched using HMMER [125] with the respective cut-off of each model. The domain functions were obtained from the GO database [126] using the PFAM2GO dataset [126].

The LAB-Secretome database was created in MySQL and the database interface was written in PHP (version5.2.7). Visualization of the protein domain composition was made using scalable vector graphics (SVG).

### Protein clustering into orthology groups (LaCOGs)

First, the 22,191 proteins in 3195 LaCOGs generated by Makarova *et.al *[1] from 12 LAB genomes were used as the basis for protein clustering. All protein sequences from 14 newly sequenced LAB genomes were searched against the Makarova LaCOG set using BlastP. The proteins that have high homology to the existing LaCOGs were then selected using a revised criterion based on the well-known COG extension rule "uniform top 3"[127]: if all the top 3 (in case of LaCOG size of 2, the top 2 hits were taken) BlastP hits of a query protein belong to the same LaCOG (LaCOG size bigger than or equals to 2), then the query protein is added to this LaCOG.

Since the above-mentioned extension was purely based on the homologs of proteins that were already included in the LaCOGs by Makarova *et al*., the specific proteins from newly sequenced species, e.g. *L. reuteri*, were not added due to the absence of the "seeding sequences" for BlastP. In order to cluster all proteins that originated from the newly sequenced genomes, a complete all-to-all Inparanoid [115] search was performed in a parallel fashion with the proteins encoded in the 14 new genomes to identify orthologous proteins. Cut-off settings of bit score 50 and sequence overlap of 50% were used. The proteins with all-to-all bidirectional-best-hit (BBH) relationship [128,129] were clustered into groups, meaning that in any such group, each member is the BBH of another member. This stringent criterion generates new cores of orthologous proteins.

Using the core ortholog clusters and the extended LaCOGs made in step one, the proteins that were not previously included in any clusters, including those proteins from Makarova LaCOGs containing only 1 member, were Blasted as queries. In this step, the revised criterion "uniform top 3" was used and new LaCOGs were made.

The newly made LaCOGs were merged with the extended Makarova LaCOGs, and the newly made ones were assigned coding numbers starting with "9" in their names, e.g. LaCOG90001, to distinguish them from the extended Makarova LaCOGs.

### LaCOG quality control

In order to check the quality of the merged LaCOGs, an iterative BlastP search was performed using the clustered proteins as queries against all the proteins that were not included in any constructed LaCOGs, using the criteria of 1E-3 and query-hit protein length ratio of 0.6, which has been tested by *Boekhorst et. al. *[130] for distant homolog identification. This iterative search found that only 13 non-clustered proteins (mostly hypothetical proteins) had a distant homolog in 11 different LaCOGs, indicating that our clustering methods have extensively included most of the proteins into possible homologous clusters.

### ORFans and proteins with only non-LAB distant homologs

The LAB proteins that could not be clustered into LaCOGs by the previously described procedures were then collected and Blasted against all completely sequences non-LAB bacterial genomes (both Gram- and Gram+ species). The same criterion of distant homolog identification [130] was utilized. Proteins that had no homologs in any other species were named "ORFans".

### Secretome LaCOG extraction

The clustering information of merged LaCOGs, proteins that have only distant homologs in non-LAB species and the ORFans was then combined with the SCL prediction made by LocateP (Table 1). Initially, only the LaCOGs that had at least half of the members with a predicted secretome SCL corresponding to (1) lipid-anchored; (2) N-/C-terminally anchored; (3) secreted by Tat- or Sec- pathway; (4) secreted via non-classical pathways, or (5) cell-wall anchored were identified as the secretome LaCOGs. Later, all other LaCOGs were manually inspected, and a few families were identified with a mixture of secretome and intracellular proteins; only the secretome proteins were added to the database. The same classification was applied to the secretome ORFans and proteins that have only distant homologs in non-LAB species. The resulting clusters of secretome proteins, the "secretome", can be further extended by similar processes when new (LAB) genome sequences become available.

Proteins that are exported by unknown mechanisms and so-called "moon-lighting" proteins (known intracellular function, but often also found on the outside of the cell) [131] were not considered as their extracellular SCL cannot be predicted.

### Manual curation

In order to obtain as accurate as possible prediction of secretome proteins and their classification into LaCOGs, we performed a throughout manual inspection on all the secretome proteins, including the ORFans and the ones included in LaCOGs. All proteins were double checked for the ORF-calling quality by the criteria combining protein length, possible alternative start (end) codon, multiple sequence alignments, protein domain composition and SCL prediction consistency.

Incorrectly chosen start codons in the original annotations were corrected based on sequence alignment with protein family members, position of putative ribosome-binding sites, and known features of signal peptides. Pseudogenes were initially identified when BLASTP analysis of the encoded proteins showed that they belong to extracellular protein families in LaCOGs, but that they represented only a fragment of the protein. By analysis of the coding region of these pseudogenes with their adjacent nucleotide sequences we could generally identify frameshifts, such that the missing protein part(s) were found to be encoded in a different reading frame. In these cases, the entire opening-reading frames were translated into protein fragments, regardless of the absence of start codons, and these protein fragments were concatenated to form new protein sequences that share high similarity to other known full-length proteins. In a few cases, ORFans were also identified as pseudogenes when they lacked a signal peptide, but otherwise contained protein domains typical of extracellular proteins.

Generally, we expected the ORFans to be real genes that represent unique functionality to the specific LAB in which they occur. However, because the average size of these hypothetical ORFs was below 100 amino acids, it is possible that some small ORFans could as well be wrongly predicted ORFs or pseudogenes. Proteins smaller than 80 amino acids containing only a Sec-type N-terminal signal sequence were removed from the set of predicted extracellular proteins, since their C-terminal part is generally too small to represent an extracellular domain. Moreover, many of such small proteins with a single predicted TM helix are now increasingly considered as small integral membrane proteins [132].

## Authors' contributions

MZ, DT and MW carried out the LaCOGs and database construction. RS performed the manual curation of the clustered proteins and DT and MM refined the LAB-Secretome database. MM and DT drafted the manuscript. Both MW and RS participated in its coordination and helped to draft and finalize the manuscript. All authors read and approved the final manuscript.

## Supplementary Material

Additional file 1 **The overview of LAB-Secretome**. Sheet S1: an overview of secretomes included in this research; sheet S2: the presence and absence patterns of the LaCOGs in 26 LAB genomes; sheet S3: the ubiquitous LaCOGs; sheet S4: the niche-specific LaCOGs; sheet S5: the species-specific LaCOGs; sheet S6: the ORFans; S7: the proteins with only distant homologs. Click here for file

Additional file 2 **False-positive SCL predictions**. The false-positive SCL predictions that were corrected using domain composition and homolog information of LaCOGs. Sheet S1: the intracellular proteins that had been wrongly predicted to be extracellular; sheet S2: the N-terminally anchored LaCOGs with C-terminal inside topology. Click here for file

Additional file 3 **The extracellular pseudogenes**. The secretome pseudogenes. The pseudogenes with wrongly annotated start/end codons were corrected and concatenated with corresponding gene neighbors. The resulting proteins seem to have homologs in various LaCOGs. The concatenated protein sequences are listed in the last column, with an "x" showing the conjunction site of each sequence. Click here for file

Additional file 4 **Interesting cases of extracellular protein families**. The distribution of binding protein families: sheet S1: Nlpc-P60 families; sheet S2: Cell surface hydrolase; sheet S3: Binding proteins. Click here for file
